# Endothelin-1 Role in Human Eye: A Review

**DOI:** 10.1155/2010/354645

**Published:** 2011-03-03

**Authors:** Serena Salvatore, Enzo Maria Vingolo

**Affiliations:** Department of Ophthalmology, University “La Sapienza”, Polo Pontino, Latina, Italy

## Abstract

Endothelin is a potent vasoactive peptide occurring in three isotypes, ET-1, ET-2, and ET-3. Through its two main receptors, endothelin A and endothelin B, it is responsible for a variety of physiological functions, primarily blood flow control. Recent evidence from both human and animal models shows involvement of endothelin in diabetes, retinal circulation, and optic neuropathies. Increased circulating levels of endothelin-1 (ET-1) have been found in patients with diabetes, and a positive correlation between plasma ET-1 levels and microangiopathy in patients with type-2 diabetes has been demonstrated. In addition to its direct vasoconstrictor effects, enhanced levels of ET-1 may contribute to endothelial dysfunction through inhibitory effects on nitric oxide (NO) production. Experimental studies have shown that chronic ET-1 administration to the optic nerve immediately behind the globe causes neuronal damage, activation of astrocytes, the major glial cell in the anterior optic nerve, and upregulation of endothelin B receptors. This paper outlines the ubiquitous role of endothelin and its potential involvement in ophthalmology.

## 1. Introduction

Endothelin 1 (ET-1) is a potent vasoconstrictor [1] peptide that is also expressed in neurons [2]. The peptide comprises 21 amino-acids with two intrachain disulfide linkages and was first isolated from the culture supernatant of porcine aorta endothelium cells in 1988 [1]. Endothelin derives from “big endothelin” a prepropeptide that is cleaved by endothelin- converting enzymes to produce mature endothelin [3]. 

Since its identification, endothelin has attracted intensive interdisciplinavy interest because of its unique profile as an endothelium-derived vasoactive factor with a powerful and characteristically long-lasting vasopressor activity. Thus, whereas cellular mechanism of endothelin action appear to be similar to classic vasoconstrictor substances such as angiotensin II and norepinephrine, the slow time course of the regulatory mechanisms of its biosynthesis and secretion resembles more that of inflammatory cytokines. These characteristics make this family of small peptides unique within the realm of intercellular mediators with cardiovascular relevance [4]. 

Endothelin is expressed in three isoforms called ET-1, ET-2, and ET-3, with slightly different amino-acid sequences and different distribution in various tissues. Accordingly, three different genes encoding the endothelins have been identified in the human, rat, and pig genome [5]. Furthermore, three ET receptor subtypes called ET_A_, ET_B_ and ET_C_ have been described.

The presence of ET-1 has been demonstrated in the human brain, pituitary gland [6], spinal cord [2], lung [7], rat bladder [6], rat kidney, feline intestinal tissue [7] and human and rat eye [8]. In rabbit, ET-1 has also been found in the tear glands [9]. ET-1 is present in the aqueous humour at concentrations several times higher than in plasma, presumably because it is secreted by the ciliary epithelium and not derived from plasma [10]. ET-3 is less ubiquitous and is found in the iris, ciliary body, and retina in some species. The presence of ET-2 in the eye has not been studied extensively. However, in the context of a possible role for this isoform in the eye pathophysiology Murata and coworkers found no evidence of ET-2 gene expression in the retina [11]. 

The vascular endothelium modulates local vascular tone by releasing relaxing factors such as nitric oxide, prostacyclin, and endothelium-derived hyperpolarizing factors as well as the potent vasoconstrictor peptide endothelin-1 [12]. Although this local regulatory system can be found in nearly all vertebrates, a great heterogeneity exists between different species, particularly in the various chemical and hormonal agonists that can stimulate the release of these endothelium-derived factors and between large and small vessels and different vascular beds [13]. 

Haefliger and coworkers [14] demonstrated the presence of both the endothelium-derived relaxing factor, nitric oxide, and the vasoconstrictor peptide endothelin in the human ophthalmic artery. These authors found that in human ophthalmic arteries, the endothelial L-arginine/nitric oxide pathway is active under basal conditions and is further stimulated by bradykinin, acetylcholine, and histamine, whereas ET1 has a potent vasoconstrictor effect. The potency of these responses suggests an important physiological role for endothelium-derived vasoactive substances in the regulation of the human ophthalmic circulation. Furthermore, a dysfunction of these endothelial mechanisms, which occurs in diabetes and hypertension (at least in peripheral arteries) may play an important role in the pathophysiology of ophthalmic complications [15].

In addition, in a subpopulation of patients with glaucoma presenting ocular vasospasms, endothelial dysfunction may represent the underlying cause or at least contribute to alterations in ophthalmic blood flow [16]. 

Vascular ET-1 is synthesized primarily in the endothelium, although it can also be produced in vascular smooth muscle cells, macrophages, leukocytes, cardiomyocytes, and fibroblasts [17]. 

In the kidney, tubular epithelial cells, mesangial cells, and podocytes are capable of ET-1 release [18]. 

Several mechanism are involved in the clearance of ET-1 from plasma, including endocytosis in the lungs, enzymatic degradation, degradation of the endothelin_B_ receptor ligand complex, and enzymatic processes in the kidney and liver [19, 20]. 

ET-1 is the most potent vasoconstrictive substance known, more potent than angiotensin II [1]. It has been shown to be highly concentrated in human atherosclerotic plaques with coronary vasospasm [21] and is also present in human plasma [22]. In addition, ET-1 mediates a wide variety of biological activities, including contraction of non vascular smooth muscle [23], thus leading, for example, to bronchoconstriction [24]. ET-1 also stimulates mitotic activity of rat vascular smooth muscle cells [25] and is thought to act as neuropeptide [2]. 

Endothelins are also associated with neuronal apoptotosis in the central nervous system. Exposure of retinal ganglion cells (RGCs) or RGC-5 cells, a transformed cell line, to ET-1 causes apoptic cell death. In addition, it was demonstrated that ET-1 enhances the glutamate-induced death of cultured retinal neurons. Furthermore Syed and colleagues [26] showed that a nonselective ET antagonist is neuroprotective in the rat retina during ischemia/reperfusion. These findings indicate that ET-1 is involved in the cell death signalling pathway(s) in retinal neurons. Interactions between ET-1 and nitric oxide synthase (NOS) have been well documented. For example stimulation of ET_B_ receptors leads to vasodilation through the formation of nitric oxide (NO), and an intravitreal injection of ET-1 enhances NO production in the optic nerve of rabbits. NOS is strongly coupled to the generation of superoxide through the uncoupling of NOS. NO can affect mitochondrial function and increase the formation of superoxide by mitochondria. Under these conditions, NO is inactivated by superoxide anion and changed into highly toxic peroxynitrite. Superoxide also causes neuronal apoptosis, and an inhibition of its formation has neuroprotective effects. Reactive oxygen species (ROS), for example, nitric oxide, superoxide, and peroxynitrite, contribute to neurodegenerative diseases including Parkinson disease and amyotrophic lateral sclerosis. 

In a recent study, Oku and coworkers also demonstrated that ET-1 causes death of retinal neurons through activation of NOS and production of superoxide anion [27]. 

Although ET-1 appears to act mainly as a local paracrine/autocrine peptide, circulating levels of endothelin seem also to have biological significance, especially in pathological states of increased serum concentration.

So far there has been no comprehensive report on the multifunctional role of ET-1 in the human eye. Therefore, the present paper is specifically addressed to illustrate ET-1 functions in the healthy and diseased eye.

## 2. ET-1 and Human Corneal Epithelium

ET-1 was found in the human corneal epithelium [28]. Interestingly, ET-1 has also been demonstrated in rabbit tear fluid [13]. Furthermore, in the rabbit ET-1 is able to induce proliferation of cultured corneal epithelial cells [29] and to promote corneal wound healing. Bek and McMillen [30] found that ET-1 is angiogenic in the rat cornea and this effect appears to be direct and dependent on activation of the ET_A_ receptor. Likewise, ET-1 might be involved in regulating growth of human corneal epithelial cells, which is important for the continuous renewal of cornea and recovery from corneal trauma and keratoplasty.

## 3. ET-1 and Retinal Circulation

It has been demonstrated that endothelins can modulate retinal pericyte contractility and, hence, retinal hemodynamics. Because of the potential importance of pericytes in maintaining normal retinal physiology and the involvement of pericyte dysfunction in diseases such as diabetes and diabetic retinopathy, studies were performed to characterize the local effects of endothelin on retinal hemodinamics in the rat eye. The retinal hemodynamic changes in response to intravitreal injections of endothelin were quantitated in diabetic and nondiabetic rats using a retinal video fluorescein angiography system. Bursell and coworkers [31] demonstrated that the retinal circulation responds directly, in a dose-dependent manner, to ET-1 injection. The vasoconstrictor activity appears to be associated primarily with the retinal arteries, whose diameter is reduced by 17% 15 minutes after 10^−7^ M ET-1 injection, compared to baseline preinfusion diameters. Correspondingly, the veins demonstrated a non significant (6%) reduction in diameter that was at least three times smaller than the arterial response. In the diabetic rat, the retinal response to ET-1 injection was approximately 10 times smaller (*P* = .01) than that observed in the nondiabetic rats. This reduction in the physiological response in diabetic animals parallels that observed in retinal pericytes cultured under high-glucose conditions and suggests that retinal pericyte function is impaired at early stages in diabetes. It has also been shown that glucose can cause an enhanced secretion of endothelin from cultured endothelial cells and that retinal pericytes demonstrate prolonged desensitization to further stimulation by ET-1 after the first application of endothelin. The findings suggest that the blunted ET-1 response observed in diabetic rats results from ET-1 secretion enhanced by glucose, endothelin receptor desensitization, or both. Although this phenomenon is still under investigation the fact that retinal blood flow is decreased at an early stage in diabetes suggests that increased ET-1 secretion and the resultant vasoconstriction at the microcirculatory level, potentially mediated by the retinal pericytes, contributes to the decreased retinal blood flow observed in diabetic animals. This may represent one of the molecular mechanisms related to the subsequent development of microvascular disease and diabetic retinopathy.

## 4. ET-1 and Diabetic Retinopathy

**Figure 1:**
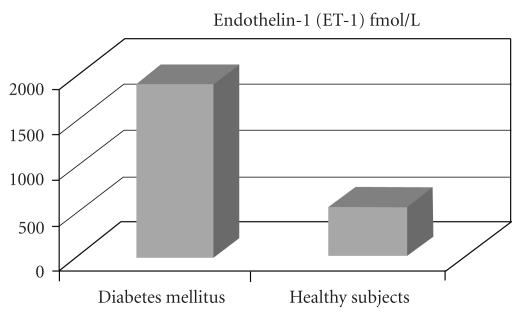
The plasma immunoreactive-endothelin concentrations were found to be greatly raised in the patients with diabetes (1,880 +/− 120 fmol/l, mean +/− SEM) compared with the healthy subjects (540 +/− 50 fmol/l, *P* less than.005). Figure adapted from Takahashi et al. [32].

Several studies support the hypothesis that endothelial dysfunction anticipates type 2 diabetes, indicating that vascular endothelial dysfunction may precede insulin resistance, although the features of insulin resistance syndrome include factors that have negative effects on endothelial function. Impaired endothelial-dependent and independent microvascular reactivity has also been demonstrated in healthy subjects with risk factors for type 2 diabetes. One important feature of endothelial dysfunction is an increased production and biological activity of the potent vasoconstrictor and proinflammatory peptide ET-1. Elevated levels of ET-1 are found in patients with type 2 diabetes, [33] (see Figure 1), and ET-1-induced reduction in insulin sensitivity may take part in the development of the metabolic syndrome. Therefore, in diabetes mellitus the disturbance in ET-1 production from vascular endothelium represents an early phenomenon rather than the result of advanced stage of the disease.

Furthermore, ET-1 may contribute to the development of endothelial dysfunction, and consequently insulin resistance, by increasing the production of reactive oxygen species, mainly superoxide anion, in the vasculature. This is mainly dependent upon activation of NADPH oxidase protein expression and activity. ET-1 levels in tissues are closely linked with reactive oxygen species (ROS) to serve as pro- inflammatory factors, and ROS are considered important factors in mechanisms underlying diabetic complications and cardiovascular derangement [34]. 

Su et al. [35] investigated at the molecular level the effects of total triterpene acids (TTAs) from Fructus Corni on early diabetic complications and whether the novel endothelin receptor antagonist CPU0213 could reverse these pathological changes via suppression of ET and inducible nitric oxide synthase (iNOS) in early diabetic retinopathy. Furthermore, they compared the effects of TTAs with those produced by blocking the receptors of advanced glycation end-products (RAGE) and iNOS by amino guanidine an advanced glycation end-products antagonist. They found that activated RAGE may accelerate the biosynthesis and release of inflammatory factors, including ET-1, ROS and iNOS. Endothelin-1 and ROS interaction results in a vicious cycle leading to mutual enhancement of vascular and cellular effects that converge to represent aetiological factors that damage macromolecules in tissues. This process may involve nuclear factor-(NF-) kB and tumor necrosis factor-*α*.

The extracellular matrix of the retina, which accumulates in retinopathy, is composed of mainly fibronectin, which undergoes alternative splicing to produce embryonic isoforms of fetal fibronectin, and increased levels of fibronectin contribute to retinopathy [36]. 

Fibronectin and its isomers are involved in endothelial cell proliferation and the increase in the extracellular matrix can be prevented by endothelin receptor antagonism by bosentan [37]. 

Diabetes produces increased microvascular permeability along with increased vascular endothelial growth factor (VEGF) mRNA expression, resulting in the deposition of hard exudates in the retina, where VEGF and ET-1 are known to interact. Thus, upregulation of the ET_A_ receptor can be considered a biomarker for the development of early retinopathy in diabetes. The endothelin receptor antagonist CPU0213 does not substantially affect fasting serum glucose, but it improves the vascular and retinal complications of diabetic rats, in agreement with its amelioration of diabetic cardiomopathy. Furthermore, TTAs reverse the abnormal upregulation of the ET system in the vasculature and the abnormal expression of the ETA receptor and iNOS mRNA in retinal tissues. Thus, TTAs are likely to exert antioxidative and antiproliferative effects on diabetic retinopathy and vasculopathy by suppression of the protein kinase C (PKC) and mitogen-activated protein kinase (MAPK) pathways and, indirectly, by suppressing the overexpression of ppET-1, ECE, ETA receptor and iNOS mRNA. Thus, the effects of TTAs resemble those of endothelin receptor antagonists and may be superior to amino guanidine in alleviating diabetic complications such as retinopathy and vasculpathy. 

Compromised vasorelaxation reduces blood perfusion in the retina, leading to upregulation of the ET system and to higher levels of ET-1 in the vasculature. This will further impair relaxation of vasculature smooth muscle because ET-1 is a very potent vasoconstrictor. More importantly, ET-1 produces proliferation of the vascular smooth muscle, increasing the thickness in the vasculature, reducing lumen size, increasing its thickness of the basement membran and thus decreasing permeability in the retina. The proliferative actions of ET-1 are likely to increase extracellular matrix and stimulate fibrosis in the retina, eventually producing proliferative retinopathy. Golubovic-Arsovska [38] found that upregulation of ET_A_ receptor is related, at least in part, to the development of preproliferative and proliferative retinopathy in clinical settings and correlates with the appearance of maculopathy and macular edema. 

The overexpression of ET-1 serves as a key biomarker for endothelial dysfunction that is likely related to superoxide generation and activated phosphatidyl 3-kinase activity [39]. 

Makino et al. [40] suggested that an increase in the basal and *α*-adrenoceptor agonist-induced release of ET-1 in diabetes can be attributed to an overexpression of the mRNA for the ppET-1. An excess of ET-1 binding to the ET_A_ receptor stimulates the PKC and MAPK pathways, which leads to phenotypic abnormalities in the genes encoding for the ppET-1, iNOS, proliferation of matrix formation, and vascular smooth muscle [41]. 

The molecular mechanisms of NOS regulation in hyperglycemia are not fully known, but recent studies point to a decisive role of an activated PKC pathway. Hyperglycaemia markedly activates the BII isoform of PKC in endothelial cells by promoting *de novo* synthesis of diacylglycerol and increasing mitochondrial superoxide production [42]. 

Diabetic retinopathy is a potentially sight-threatening complication that develops in nearly all patients with diabetes. Ocular tissues, for example, vascular and extravascular sites in the retina are sources of ET-1, and ET-1 contributes to abnormal retinal hemodinamics in diabetic retinopathy. Results from several studies on streptozocin-induced diabetes in rats have suggested a role of ET-1 in the pathogenesis of diabetic retinopathy. An interaction between ET-1 and VEGF has also been reported, and preliminary positive results of treatment with endothelin receptor blockers have raised interest in these substances as potentially therapeutic agents. Most of the studies on the role of ET-1 in the pathogenesis of diabetic retinopathy and on the importance of endothelin blockers in the treatment of this serious complication have been done in animal models. Nevertheless, they provide sufficient evidence that strongly links ET-1 to the pathogenesis of diabetic retinopathy.

## 5. ET-1 and Retinitis Pigmentosa

Retinitis pigmentosa (RP) is a group of clinically and genetically heterogeneous retinal degenerations characterized by chronic progressive loss of rod and cone photoreceptor function [44]. The damage is actually explained with an invalid gene product or protein causing a metabolic wrong step in the phototransduction process. Different inheritance patterns of tapetoretinal degenerations were described: autosomal dominant, recessive and X-linked, all of them associated with point mutations and intragenic micro deletions as well as other molecular defects within over forty-five different RP and loci [45]. Hemodynamic studies have demonstrated that RP is associated with a reduction in retinal and choroidal blood flow. Retinal hemodynamic impairment is also present in early stages of RP and among the various hypotheses advanced on the causative factors involved in this impairment the ET-1-mediated vasoconstriction is a likely candidate.

ET-1 has been shown to be expressed in the retina, including the photoreceptors, the inner plexiform layer and the ganglion cell layer [46]. The choroid, the vascular smooth muscle in retinal blood vessels and choriocapillaris have also been shown to contain receptors for ET-1.

Secretion of ET-1 by the retinal pigment epithelium (RPE) could target receptors on the apical (photoreceptors) or basal (choroid) sides. Activation of ET receptors in the retinal or choroidal vasculature may be important in regulating blood flow at this region. Haemodynamic studies have demonstrated that RP is associated with a reduction of retinal and choroidal blood flow [47] even in early stages of RP.

**Figure 2:**
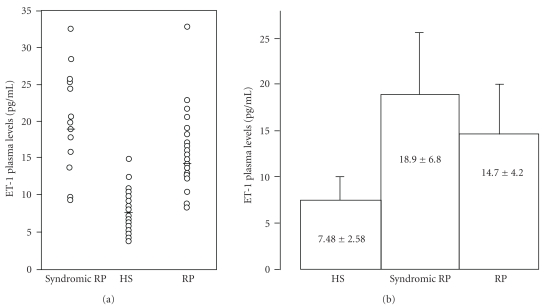
(a) Scatter plot of ET-1 plasma values in patients with retinitis pigmentosa (RP), syndromic retinitis pigmentosa (syndromic RP), and healthy subjects (HS). (b) Mean (M ± SD) plasma ET-1 levels in patients with RP, syndromic RP, and HS. Figure adapted from Vingolo et al. [43].

These vascular abnormalities might be correlated with the increased plasma ET-1 concentration that was found in two studies on RP patients [43, 48] (Figure 2). Although these data require further investigation in a larger population of patients, these studies open the possibility that antiendothelin drugs could result therapeutically useful in RP administered either alone or in association with treatments directed to improving tissue oxygenation such as hyperbaric oxygen therapy [49].

## 6. Endothelin-1 and the Optic Nerve Head

**Figure 3:**
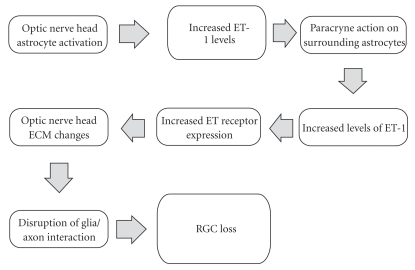
Potential mechanism of endothelin (ET) and ET receptor-mediated actions causing retinal ganglion cell (RGC) loss. Optic nerve astrocytes may become activated via ET actions, leading to extracellular matrix (ECM) changes in the optic nerve head and eventual RGC loss. Figure adapted from Chauhan BC [50].

Open angle glaucoma is the most common optic neuropathy causing retinal ganglion cell (RGC) soma and axon loss, optic nerve head (ONH) structural loss, and visual field damage. Elevated intraocular pressure (IOP) is the most potent risk factor known for causing glaucomatous damage. Decades of experimental and clinical research consolidated the notion that lowering IOP has a favourable impact in the majority of patients with glaucoma. This has been confirmed in randomized clinical trials that included untreated control arms [53]. How IOP can lead to structural damage and produce clinical glaucoma is not fully understood. Much evidence points to the ONH, consisting of RGC axons, blood vessels, connective tissues and glia, as the primary site of damage in glaucoma [54]. It is likely that the microenvironment in the ONH reacts to stressors such as IOP and other potential IOP-dependent or IOP-independent factors, such as ischemia, to ultimately cause RGC axonal damage (Figure 3). The potential of endogenous vasoconstrictors to cause ischemic insult in the ONH was proposed many years ago and endothelin represents one vasoconstrictor agent with a possible role in glaucoma and other neurodegenerative diseases (Figure 3). An impairment of the anterior optic nerve microcirculation has been suspected to contribute and/or to be a causal factor in a variety of optic neuropathies. Thus, endothelins that produce dose-dependent vasoconstriction in various vascular beds, including the anterior optic nerve microvasculature, become likely candidates able to produce neural damage. Indeed, these peptides have been shown to produce localized vasoconstriction when injected directly into perivascular cerebral tissues *in vivo* and to result in regional ischemic damage of the brain nervous tissue [55]. 

**Figure 4:**
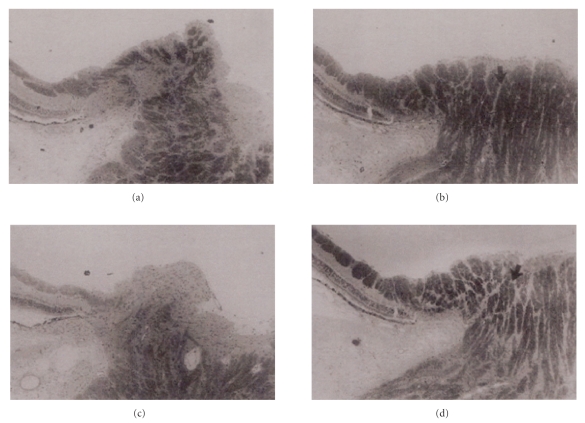
Light microscopic view of two pairs of optic nerves (toluidine blue stain). Optic nerves subjected to endothelin-1 during 8 weeks ((a) and (c)) showed a loss of myelin (dark areas) and a gliosis of the prelaminar portion of the optic nerve compared to the contralateral eyes ((b) and (d)). Arrows point to myelinated nerve fibers in the control eyes ((b) and (d)). Figure adapted from Orgul et al. [51].

Increased plasma ET-1 levels have been described in normal tension glaucoma patients, although this finding was not confirmed in every study dealing with normal tension glaucoma patients or in studies with high tension glaucoma patients. On the other hand, the fact that aqueous ET-1 concentration is increased in primary open-angle glaucoma and in animal models of glaucoma underscores the possible contribution of endothelin to the pathogenesis of primary open angle glaucoma. Furthermore, chronic administration of ET-1 has been shown to produce an optic neuropathy similar to glaucoma. In the rabbit animal model of glaucoma Orgül et al. [51] assessed optic nerve blood flow after local administration of endothelin-1 *in vivo*. In addition, the effect of optic nerve ischemia was monitored by means of a confocal scanning laser ophthalmoscope. Administration of endothelin-1 to the anterior optic nerve region induced a significant decrease in local blood flow of approximately 38% compared to the contralateral eye. Multivariate analysis disclosed a small, but statistically significant change in optic nerve morphology, as measured with a confocal scanning laser ophthalmoscope, after 8 weeks of local administration of endothelin-1, compared to the control eye. These changes were consistent with an optic nerve cupping and a decrease in optic nerve rim volume. Histologic analysis showed loss of myelin and gliosis of the prelaminar portion of the optic nerve in optic nerves subjected to endothelin-1 for 8 weeks (Figure 4). Blood flow and morphologic changes were independent of changes in intraocular pressure. 

**Figure 5:**
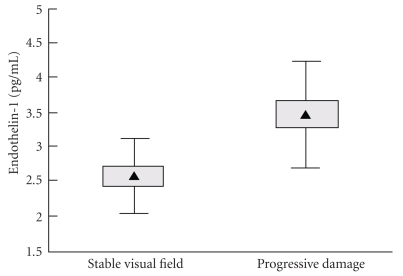
Plasma concentration of ET-1 in patients with glaucoma with and without visual field progression (triangle = mean; box = standard error od the mean; whiskers = standard deviation). Plasma levels of ET-1 at the end of the observation period were significantly higher in patients with progressive damage (3.47 (0.75) pg/ml; range 2.34–5.17 pg/ ml) compared with those with stable (2.60 (SD 0.54) pg/ml; range 1.91–3.45 pg/ml) visual fields (ANOVA: *F*  (1,29) = 13.94281; *P* = .0008). This difference was still significant after controlling for the interaction of sex and including age and MBP as covariates into the model (ANCOVA: *F*  (1,25) = 14.95; *P* = .0007). Sex had a borderline significant effect (ANCOVA: *F*  (1,25) = 4.25; *P* = .0497), but the interaction between sex and disease progression was not significant (ANCOVA: *F*  (1,25) = 1.84; *P* = .19). Figure adapted from Emre et al. [52].

In healthy young humans, the circulating levels of endothelin are low [56]. In pathologic conditions such as an ischemic cerebrovascular insult, the plasma level of endothelin-1 has been reported to be elevated [57]. Emre and coworkers [52] found increased plasma level of ET-1 in primary open-angle glaucoma patients with progressive damage when compared with primary open-angle glaucoma patients with stable visual fields, this difference was independent of sex, age, and mean blood pressure. Furthermore, they found that values obtained among patients with a stable visual field were above the reference values established in their laboratory (Figure 5). A possible explanation would be that ET-1 may contribute to the initiation of the damaging processes in glaucoma, but then remain increased as a consequence of the damage. 

Endothelin may also directly regulate the blood flow within the optic nerve [58]. The ocular circulation is particularly sensitive to changes in local ET-1 concentration, even at doses that do not affect the systemic haemodynamics or flow velocity in the ophthalmic artery [59]. In some individuals with primary open-angle glaucoma, higher-than normal plasma and asqueous humor concentrations of ET-1 have been observed [60–63]. This high concentration of ET-1 has been associated with reduced blood flow in the posterior ciliary arteries that supply the optic nerve vasculature. Peripheral vascular response to ET-1 is also altered in some individuals with glaucoma. Intravitreal injections of ET-1 into the rabbit eye produce marked effects on the anterior ciliary circulation, as well as constriction of the retinal vasculature. With ET-1 administration to the retrobulbar perineural space, localized constriction in the posterior ciliary arterial circulation has been produced in both the rabbit and primate eye [64].

## 7. Endethelin-1 and Retinal Detachment

ET-1 is found in the photoreceptor inner segments of the human retina where it may play a role in neuromodulation or neurotransmission [65, 66]. 

ET-1 immunoreactivity is present on the cell body of the astrocytes and both ET-1 mRNA and ET-1 immunoreactivity have been found in the retinal pigment epithelium (RPE) cells. Elevated subretinal fluid (SRF) and vitreous immunoreactive ET-1 (IR-ET-1) [67] levels were associated with RD (retinal detachment) and proliferative vitreoretinopathy (PVR) [68]. 

IR- ET-1 was localized in the cellular and stromal components of PVR membranes where, ETA and ETB receptor expression has also been demonstrated. 

The significant correlation found between SRF-IR-ET-1 and plasma IR-ET1 suggests that intraocular IR-ET1 derives in part from systemic circulation, but the slope of the correlation, always greater for the PVR group, is consistent with enhanced access of plasma proteins in the eye due to a disruption of the blood-ocular barrier. The increased difference of SRF- plasma IR-ET1 in the PVR group may also suggest a local ET-1 production in PVR.

Endothelin could play a role in photoreceptor synaptic transmission, and this would require thight control of the endothelin extracellular concentration. Modulation of synaptic transmission might affect photoreceptor survival, perhaps by regulating glutamate release [69]. 

RPE and glial cells are the main contributors to membrane formation and contraction in PVR. The possibility that RPE plays a role in endothelin-mediated photoreceptor survival cannot be excluded because this retinal layer contains ET-1, prepro-ET-1, and ETA immunoreactivities [70]. 

ET-1 may also act as growth factor for astrocytes, inducing DNA synthesis and proliferation [71]. Astrocytic proliferation together with an excessive secretion of ET-1 has been reported in cerebral focal ischemia *in vivo *[72]. Sasaki et al. [73] demonstrated that ET-1 specifically stimulated the efflux of glutamate via ETB receptors from cultured rat astrocytes, suggesting that ET-1 may esacerbate neurodegeneration. Infusion of ETB selective antagonists attenuates the increase in astrocytes after injury of the brain cortex, indicating that induction of reactive astrocytes depends on the activation of ETB receptors [74]. Reactive gliosis has been suggested as a clinically significant limiting factor in the recovery of vision after RD. Excess of ET-1 released by injured glial cells can be compensated by scavenging ETB receptors, [75] and it has been suggested that blockade of these receptors after central nervous system injury might modulate glial scar formation, providing a more permissive substrate for neural survival and regeneration [76]. Roldán-Pallarés et al. [77] investigated the relationship between visual acuity (VA) and SRF-IR-ET1 levels in RD. They concluded that VA was inversely correlated with SRF- IR-ET1 levels. Nevertheless, the highest negative correlation between postoperative VA and VA difference and the SRF-IR-ET1 levels was found in the group with proliferative complication of retinal detachment (PVR). These findings support the idea of performing a primary vitrectomy at early stages of pathology in RD to eliminate the intraocular peptide and perhaps associate a pharmacologic therapy in RD, more importantly in PVR. 

ET-1 can modulate anterograde fast axonal transport, which is essential for maintaining synaptic function and neuronal survival [78]. 

Recently experimental evidence that stimulation of endothelinergic receptors may modulate photoreceptor survival and glial activation has been provided [79].

## 8. Conclusion

Antiendothelin monoclonal antibodies as well as receptor antagonists BQ-123 (ETA selective, peptidic) and Ro 46-2005 (nonselective, nonpeptidic) have been repeatedly shown to ameliorate ischemic and cyclosporine-induced acute renal failure. Chronic administration of FR 139317, another ET_A_ selective antagonist, was effective in preventing progressive proliferative renal disease and associated hypertension in a rat model of chronic glomerulonephropathy induced by surgical renal mass reduction. The blockade of endothelin action by neutralizing antibodies or BQ-123 has been shown to reduce the extent of experimental acute myocardial infarction in rat, rabbit, and dog. The peptidic ETA selective antagonist BQ-485 is effective in preventing delayed cerebrovascular spasm in a dog model of subarachnoid hemorrhage. In a similar model in the rabbit, the nonpeptidic antagonist Ro-47-0203 reverses delayed vasospasm. Furthermore, endothelin receptor antagonists lower basal blood pressure in spontaneously hypertensive and spontaneously hypertensive strokeprone strains of rats as well as in sodium-depleted squirrel monkeys, suggesting a possible role for endothelins in maintaining blood pressure control under certain conditions and in the development of genetically determined hypertension. The antagonist not only reduces blood pressure but also prevents secondary renal disease observed in DOCA/salt-treated, spontaneously hypertensive rats with malignant hypertension.

ECE (endothelin-converting enzyme) represents another target for pharmacological intervention on the endothelin system because ECE appears to be a metalloprotease that has a strict substrate specificity. A selective inhibitor for the enzyme can presumably inhibit the production of active endothelins in a highly fast manner. Unfortunately, this avenue of research has been severely hampered in the past because of the elusive molecular nature of ECE itself. However, now that ECE has been apparently purified to near-homogeneity, this major component of the endothelin system should soon be revealed at the molecular level.

Endothelin-1 itself could be the target molecule of new therapeutic drugs. In a recent study, Scorza et al. [80] analyzed the influence of aminaphtone, a 4-aminobenzoic acid derivative clinically used for the treatment of capillary disorders, on ET-1 protein production, pre pro endothelin (PPET-1) gene expression and ECE activity in human ECV304 cells after incubation with physiological concentrations of interleukin-1*β* (IL-1*β*). ECV304 cells originate from endothelial cells of human umbilical vein by spontaneous transformation and have been widely used for *in vitro* studies of the endothelium and ET-1 pathways, since they are able to produce ET-1 and express the mRNA and the *α*-isoform of ECE. They demonstrated that addition of different concentrations of aminaphtone to ECV304 cells reduces production of ET-1 and downregulates PPET-1 gene expression in a concentration-dependent manner. Moreover they observed that aminaphtone at a concentration of 6 *μ*g/mL, which roughly represents the peak plasma concentration reached after oral administration of 75 mg of the drug, reverts production of ET-1 to baseline values [80]. This may encourage clinical trials on the efficacy of this molecule in the downregulation of ET-1 levels in eye disorders. Development in the near future of nonpeptidic receptor antagonists with even higher potency and specific ECE inhibitors shoud further facilitate the understanding of the biological role of endothelins in health and disease. Those new biological insights should provide renewed promise for the progress toward this novel target for therapeutic intervention, which may include ocular diseases for which no effective drug treatment is currently available.
